# Variation in farming damselfish behaviour creates a competitive landscape of risk on coral reefs

**DOI:** 10.1098/rsbl.2024.0035

**Published:** 2024-05-29

**Authors:** Catherine E. Sheppard, Lisa Boström-Einarsson, Gareth J. Williams, Dan A. Exton, Sally A. Keith

**Affiliations:** ^1^ Lancaster Environment Centre, Lancaster University, Bailrigg, Lancaster LA1 4YQ, UK; ^2^ School of Ocean Sciences, Bangor University, Menai Bridge LL59 5AB, UK; ^3^ Operation Wallacea, Wallace House, Old Bolingbroke, Spilsby PE23 4EX, UK

**Keywords:** landscape of risk, competition, territoriality, behavioural variation, spatial distribution

## Abstract

Interspecific interactions are fundamental drivers of animal space use. Yet while non-consumptive effects of predation risk on prey space use are well-known, the risk of aggressive interactions on space use of competitors is largely unknown. We apply the landscape of risk framework to competition-driven space use for the first time, with the hypothesis that less aggressive competitors may alter their behaviour to avoid areas of high competitor density. Specifically, we test how aggressive risk from territorial algal-farming damselfishes can shape the spatial distribution of herbivore fish competitors. We found that only the most aggressive damselfish had fewer competitors in their surrounding area, demonstrating that individual-level behavioural variation can shape spatial distributions. In contradiction to the landscape of risk framework, abundances of farming damselfish and other fishes were positively associated. Our results suggest that reef fishes do not simply avoid areas of high damselfish abundance, but that spatial variation in aggressive behaviour, rather than of individuals, created a competitive landscape of risk. We emphasize the importance of individual-level behaviour in identifying patterns of space use and propose expanding the landscape of risk framework to non-predatory interactions to explore cascading behavioural responses to aggressive risk.

## 1. Introduction

Human-induced environmental change is altering the behaviour and spatial distribution of animals worldwide [1]. These behavioural shifts can cascade through ecosystems to affect species persistence, ecosystem services and resilience under climate change [1]. The abiotic drivers of behavioural and spatial patterns are well-known, however, biotic factors also play a role. Non-consumptive effects of predators on the spatial distribution of prey are well established and encompass the behavioural responses of prey to predation risk [2,3]. Interspecific competition is also a fundamental driver of spatial distribution in ecological communities [4]. Yet how the perceived risk of encountering aggressive competitors shapes spatial distribution in less aggressive species is unknown. We can explore competition-driven space use through the ‘landscape of risk’ framework [5].

The landscape of risk typically represents spatio-temporal patterns of predation risk [5]. However, similarly, the risk of encountering aggressive competitors may shape space use in less aggressive species. Competitors may avoid aggressive interactions by changing their behaviour, such as avoiding areas of high competitor density, helping them to navigate their environment at a lower risk of encountering aggressive competitors. Expanding the landscape of risk framework to competitive interactions provides a useful tool to explore these spatial and behavioural cascades.

Coral reef fish communities present an excellent system to study competition-driven space use in response to aggressive risk. Many species demonstrate clear site attachment and aggressive and territorial behaviour [6–8]. One such group of fish that is thought to influence the spatial distribution of reef inhabitants is the territorial farming damselfish. Aggressive behaviour by farming damselfish is expected to drive away herbivores and suppress herbivory inside damselfish territories, increasing turf algal cover as a result [7]. However, evidence of this effect is mixed. Experimental removal of farming damselfish, giving access to roving herbivores, has found both subsequent reductions in algal biomass [9] and no effect on benthic communities within territories [10]. Feeding by surgeonfish has even been found to decrease upon removal of farming damselfish [11]. In addition to their potential effects on herbivory, farming damselfish abundance can influence coral predation rates [12] and juvenile parrotfish recruitment [13]. The aggressive behaviour of farming damselfish has clear cascading effects on reef fish behaviour. One way to better understand their impact is to look at how they shape the space use of reef fishes.

Based on the landscape of risk framework, we expect a negative association between the abundance of farming damselfish and other reef fishes, as fish avoid areas of coral reef with greater aggressive risk. We explore how interspecific competition and aggressive risk by farming damselfish may shape the spatial distribution of the wider reef fish community on coral reefs. Specifically, we examine the spatial distribution and variation in aggressive behaviour by farming damselfish, which we term the competitive landscape of risk, alongside the spatial distribution of other reef fishes.

## 2. Material and methods

### (a) Field methodology

**Figure 1.**
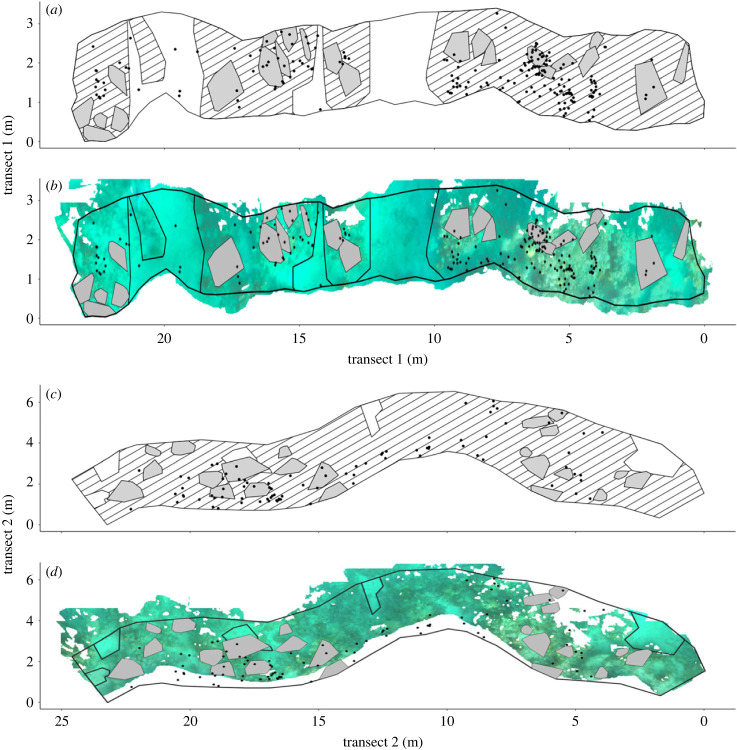
Transect maps (*a*,*b*: transect 1, *c*,*d*: transect 2). Hashed boxes represent hard substrate, grey areas show individual *Stegastes* spp. territories, points show location of non-*Stegastes* reef fishes. Subplots *b* and *d* show underlying orthomosaics. Note belt transects were 2 m wide and 25 m long, however as surveys followed the reef line, figure axes represent absolute position.

Data were collected between 13 July and 1 August 2022 at Coral View reef, Utila, Honduras (N 16.08823274, W −86.91094506), across two belt transects 25 m long × 2 m wide (separated by greater than 10 m; 100 m^2^ total) at approximately 5 m depth (figure 1). The territories of all adult damselfish of the species *Stegastes diencaeus* (*n* = 26) and *S. planifrons* (*n* = 22), hereafter *Stegastes* spp., within the belt transects were tagged with identification numbers. *Stegastes diencaeus* and *S. planifrons* were the most abundant damselfish species in the area and exhibited similar aggressive territorial behaviours (per. obs.). Although territories are used for both cultivation of turf and egg protection, no evidence of eggs or nesting behaviour was observed.

### (b) Territory mapping

We mapped the territories of *Stegastes* spp. within our belt transects using methods similar to Robles *et al*. [14]. We attached a GoPro HERO camera to a stand 1.5 m above the focal territory such that the camera had a field of view of 1.5 m × 2 m, with a visible 30 cm scale. Focal individuals were recorded for 15 min, discarding the first 5 min as an acclimation period [14]. Territory recordings were taken after behavioural videos to avoid disruption caused by placing a frame over a territory. To estimate territory size, 21 screengrabs taken approximately every 30 s across a 10 min period were imported into ImageJ [15]. We recorded the position of the focal individual as a point on each screengrab and calculated the minimum convex polygon around all points (electronic supplementary material, figure S1). In *S. diencaeus*, territory size may correlate with body size when a broad size range is observed (PT McDougall & DL Kramer 2007, unpublished observations), however, effects may not be seen over a smaller range [16]. Individuals must be caught to accurately measure *Stegastes* body size (e.g. [16,17]), which may disrupt territorial behaviour and was therefore judged unnecessary for this study.

### (c) Behavioural observations

To explore the landscape of risk around farming damselfish territories, we recorded the aggressive response of focal *Stegastes* spp. in terms of chases of reef fishes that moved through their territory, known as intruders. This measure of aggression is widely used in studies of territorial farming damselfish [14,18,19]. Each focal individual was recorded once for 30 min using GoPro HERO cameras between 07:00 and 15:00, discarding the first 5 min as an acclimation period from diver/snorkeller presence [20]. Cameras were placed 1–2 m from the focal individual's territory such that the focal individual and intruders could be observed. Previous studies recorded mean territory sizes of 1.08 and 2.83 m^2^ for *S. planifrons* and sister species *S. adustus*, respectively [21]. Therefore, the order in which focal individuals were recorded was strategically randomized such that individuals recorded at the same time were at least 3 m from each other to assure independence. Analysis of behavioural videos was undertaken in BORIS v. 8.6.2 [22]. We recorded chase behaviours associated with aggressive response, defined as accelerated swimming movements of the focal individual towards intruders. Intruders that did not elicit an aggressive response were also noted. These were identified as any non-*Stegastes* fish that entered the focal individual's territory, which was visually estimated before analysis (electronic supplementary material, figure S2). Intruders were identified to the family level.

### (d) Reef and fish surveys

Reef fishes were surveyed on SCUBA following a standardized protocol (electronic supplementary material) using a diver-operated stereo-video system (SVS), allowing for accurate measurement of fish position [23]. Belt transects were surveyed five times, with each survey spaced at least 3 h apart to reduce the likelihood of repeat samples of roving individuals. Coordinates of two reference points for each fish along the transect were also recorded. These included strategically placed golf balls and visual landmarks along the reef, such as distinct corals and rock formations.

We used structure-from-motion underwater photogrammetry to construct orthomosaics of belt transects [24,25], on which to map *Stegastes* spp. territories and non-*Stegastes* reef fish locations (electronic supplementary material). Orthomosaics were imported into QGIS Desktop v. 3.28.2 [26], on which reference points corresponding to the same reference points in the SVS data were plotted as a multipoint shapefile layer. X and Y coordinates of reference points were then extracted and used for coordinate transformation (electronic supplementary material).

### (e) Statistical analysis

Data manipulation and statistical analyses were conducted in QGIS and R v. 4.2.3 [27]. In QGIS, we plotted the minimum convex polygons of 45 focal *Stegastes* spp. alongside 285 reef fishes on our orthomosaics (figure 1*a*). Buffers of 1 m and 0.5 m were drawn around focal *Stegastes* spp. territories (figure 1*b*) and the number of non-*Stegastes* fish within the territories and buffer combined were counted. These buffers were chosen based on mean territory size (*S. diencaeus*: 0.55 ± 0.25 m^2^; *S. planifrons*: 0.29 ± 0.13 m^2^) and that farming damselfish chases are typically shorter (less than 0.5 m). The number of *Stegastes* spp. whose territories overlapped with the buffers was counted to calculate abundance of *Stegastes* spp. within buffers. Due to the prolific abundance of *Stegastes* spp. at our study site, it was not possible to draw buffers containing hard substrate but no *Stegastes* spp.

We calculated aggression metrics for each focal *Stegastes* spp. based on the proportion of non-*Stegastes* intruders that were chased, placing individual-level aggression on a scale of 0 to 1. Individual aggressive response towards heterospecific intruders differed between species (*S. diencaeus*: mean ± s.d. = 0.63 ± 0.28; *S. planifrons*: 0.41 ± 0.15). However, as our study focused on the effect of individual-level behaviour across *Stegastes* spp., and both species exhibited wide within-species variation in aggressive response (*S. diencaeus*: range = 0.10–1.00; *S. planifrons*: 0.19–0.76), species was not included in further analysis. Aggression metrics were calculated for all non-*Stegastes* intruders, and herbivorous and non-herbivorous non-*Stegastes* species separately. All fish were categorized as herbivorous or non-herbivorous according to FishBase [28]. To check for temporal variation in aggressive response, Spearman's rank tests were conducted between individual-level aggression and time of recording. No correlation between the two variables was found (*r* = 0.08, *p* = 0.60), therefore time of recording was not included in further analysis.

Using the brms package [29] implemented in STAN [30], we ran Bayesian models with a negative binomial distribution to model counts of non-*Stegastes* fish against individual-level aggression and *Stegastes* spp. abundance. Bayesian models ran for 5000 iterations, with a warm-up of 1000 iterations over four chains. Weakly informative priors were used and transect ID included as a grouping factor to account for spatial dependence. As the territory and buffer area differed between focal *Stegastes* spp., the area within which fish were counted was included as an offset. Using offsets as opposed to densities is advantageous as fitted values and confidence intervals are always positive yet heterogeneity in survey area is accounted for [31]. This also accounted for survey area differences resulting from buffers being truncated when they extended beyond the transect window. As transects were 2 m wide, this was unavoidable, and most buffers were affected. Counts of herbivores and non-herbivores were modelled separately to explore whether *Stegastes* spp. affect the spatial distribution of dietary groups differently. Bayesian models were visually validated for fit and convergence using graphical posterior predictive checks, trace and density plots and Gelman–Rubin convergence diagnostic (R-hat) [32]. To reduce the number of divergent transitions to below 20 for all models, the adapt delta control parameter was increased to 0.95. All models had R-hat values of 1.00 and effective sample sizes over 1000 signifying good model convergence. We checked for highly influential data points using leave-one-out cross validation (LOO). Pareto *k* values above 0.7 are considered highly influential [33].

## 3. Results

**Figure 2.**
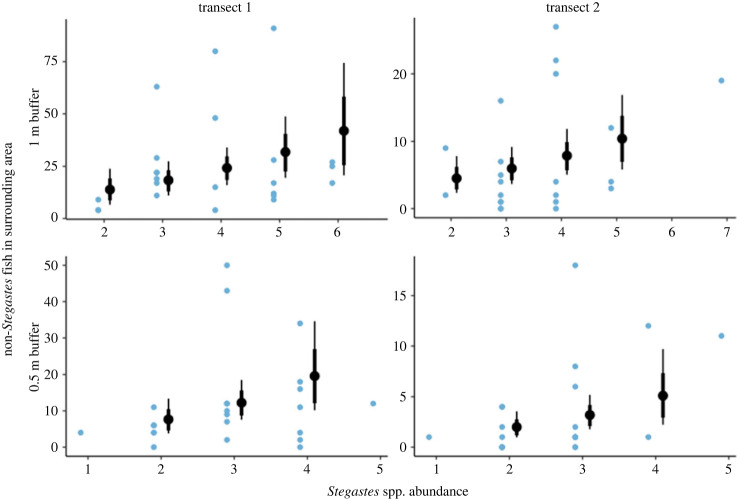
The number of non-*Stegastes* fish within *Stegastes* spp. territories and surrounding area (1 m and 0.5 m buffer) increase with increasing abundance of *Stegastes* spp. Each column shows data from a different transect, the identities of which were included as grouping factors. Blue points represent raw data. Black points and interval lines represent the expected posterior predictions and confidence intervals (80 and 95%) from Bayesian models using mean values of *Stegastes* spp. aggression and measurement area.

The number of non-*Stegastes* fish within 1 m and 0.5 m buffers increased with increasing *Stegastes* spp. abundance (1 m: *β* = 0.28, 95% CI = 0.04 to 0.53; 0.5 m: *β* = 0.47, 95% CI = 0.06 to 0.90; figure 2). When split by dietary group, the number of non-*Stegastes* non-herbivorous fish within 1 m and 0.5 m buffers increased with increasing abundance of *Stegastes* spp. (1 m: *β* = 0.35, 95% CI = 0.08 to 0.63; 0.5 m: *β* = 0.55, 95% CI = 0.09 to 1.03; electronic supplementary material, figure S3). The association between the number of non-*Stegastes* herbivorous fish within 1 m and 0.5 m buffers and *Stegastes* spp. abundance was weakly positive (1 m: *β* = 0.14, 95% CI = −0.14 to 0.42; 0.5 m: *β* = 0.22, 95% CI = −0.24 to 0.69; electronic supplementary material, figure S4).

**Figure 3.**
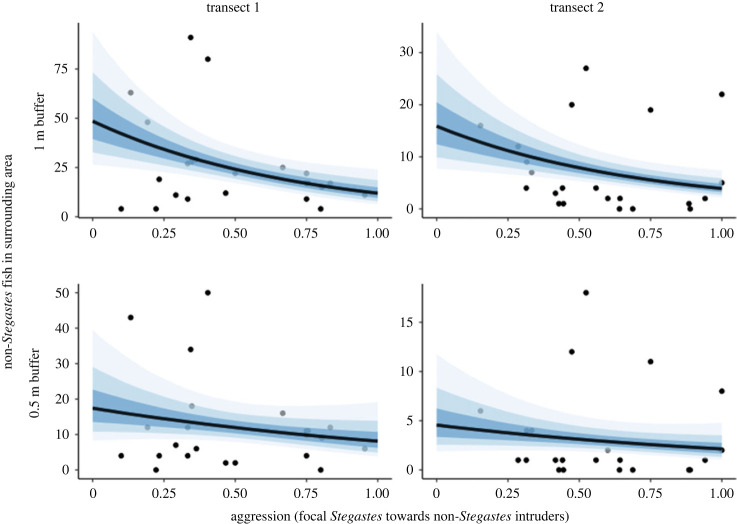
The number of non-*Stegastes* fish within focal *Stegastes* spp. territories and surrounding area (1 m and 0.5 m buffer) declines with increasing individual-level aggression of focal *Stegastes* spp. towards intruders (1 m: *β* = 0.28, 95% CI = 0.04 to 0.53; 0.5 m: *β* = 0.47, 95% CI = 0.06 to 0.90). Columns show data from two transects, the identities of which were included as grouping factors. Points represent raw data. Trend line and shaded areas represent the expected posterior predictions and confidence intervals (50, 80 and 95%) from Bayesian models using mean values of *Stegastes* spp. abundance and measurement area. Aggression is measured as the proportion of intruders into the focal territory that were chased.

More aggressive *Stegastes* spp. had fewer non-*Stegastes* fish within a 1 m and 0.5 m buffer around their territory (1 m: *β* = −1.41, 95% CI = −2.50 to −0.33; 0.5 m: *β* = −0.77, 95% CI = −2.07 to 0.57; figure 3). More aggressive *Stegastes* spp. had fewer non-*Stegastes* non-herbivorous fish within a 1 m buffer (1 m: *β* = −1.55, 95% CI = −2.80 to −0.34; 0.5 m: *β* = −0.57, 95% CI = −2.03 to 0.87; electronic supplementary material, figure S5). There was little association between individual-level aggression and number of non-*Stegastes* herbivorous fish within a 1 m and 0.5 m buffer (1 m: *β* = −0.23, 95% CI = −1.14 to 0.73; 0.5 m: *β* = −0.31, 95% CI = −1.61 to 1.00; electronic supplementary material, figure S6). Both *S. diencaeus* and *S. planifrons* demonstrated wide variation in individual aggressive response towards heterospecific intruders (range: 0.10–1.00 and 0.19–0.76 respectively). No influential points were found in any model (Pareto *k* value > 0.7).

## 4. Discussion

We applied the landscape of risk framework to demonstrate that between-individual variation in aggressive behaviour by territorial *Stegastes* spp. may shape the spatial distribution of coral reef fishes. More aggressive *Stegastes* spp. had fewer fishes near their territories than less aggressive individuals, suggesting that fish may avoid areas of reef occupied by more aggressive individuals. This could be a learned behaviour from previous encounters or recognition of stronger threat signals given by more aggressive individuals. In contradiction to the landscape of risk framework [34], the abundances of *Stegastes* spp. and other fishes were positively correlated. This may result from clustering of reef fishes around live coral and hard substrate (figure 1). Our results suggest that reef fishes do not simply avoid areas of high *Stegastes* spp. abundance but may avoid areas defended by more aggressive individuals.

Between-individual behavioural variation can have profound effects on interspecific interactions and community dynamics [35–37]. Yet landscapes of risk typically focus on behaviour at the population or species level, relying instead on spatial variation created by patterns in distribution or density [3]. *Stegastes* spp. are prolific in the Caribbean [16,21] and occupy large areas of coral reefs, meaning there is little spatial pattern in their distribution beyond being reliant on the hard substratum (figure 1). However, both *S. diencaeus* and *S. planifrons* demonstrated wide variation between individuals in aggressive response to heterospecific intruders, creating spatial variation in aggressive competitive risk, which we term a competitive landscape of risk. We identified that between-individual variation in *Stegastes* spp. aggressive response may play a role in shaping fish distribution across reefs, adding to the growing pool of evidence of the ecological importance of individual-level behaviour [35]. We demonstrate that incorporating between-individual behavioural variation may greatly improve our understanding of spatial patterns in response to risk [38].

It is generally assumed that heterospecific aggression is greater with increased resource overlap [39]. It is therefore reasonable to expect that the response to aggressive risk be influenced by the degree of resource overlap. However, *Stegastes* spp. aggression showed little association with the abundance of non-*Stegastes* herbivores. Instead, there was a weakly positive association between the abundances of *Stegastes* spp. and herbivores. Our results support previous findings that aggressive interactions with farming damselfish may not affect herbivore abundance and subsequent herbivory rates [10,11]. Instead, herbivores may be attracted to resources within damselfish territories and the surrounding area. The farming behaviours of damselfish modify algal composition within their territories, increasing epiphyte load and cover of palatable turf algae [40], which may attract herbivores despite the increased aggressive risk. This unexpected relationship between herbivore and *Stegastes* spp. abundance further demonstrates the complexities in the spatial distribution of herbivores.

Contrastingly, more aggressive *Stegastes* spp. had less non-herbivorous fishes in the area surrounding their territory. This finding suggests that non-herbivorous fishes responded more strongly to aggressive behaviour by farming damselfish than herbivores, contrary to expectation. This finding is unlikely due to non-herbivores reacting more strongly to aggressive risk, and instead likely the result of herbivores being attracted to *Stegastes* spp. territories. However, damselfish abundance has previously been found to influence the behaviour of non-herbivores, such as reduced coral predation [12]. Therefore, the effects of aggressive risk by farming damselfish on the spatial distribution of non-herbivorous fishes cannot be overlooked.

Aggressive risk by territorial farming damselfish has the potential to shape fish distribution across vast areas of coral reef. Farming damselfish is widely considered as key drivers of coral reef benthic composition [7,41–43]. Therefore, changes to spatial distribution and community composition of reef fish driven by aggressive risk may have cascading consequences to coral reef health [44]. Just as predation risk drives prey space use and subsequent effects on ecosystem health, aggressive risk between competitors may shape spatial variation in multiple critical functions of coral reefs by altering the spatial distribution of fishes (for a review of core coral reef functions, see [45]).

Competition-driven habitat selection between pairs of species has been well-studied [46–49] and demonstrates a general trend of more aggressive species forcing subordinate competitors into less profitable habitats. However, few attempts have been made to model this interaction spatially (however see [49]). We applied the landscape of risk framework to demonstrate that aggressive interactions between competitors may shape their spatial distribution. Furthermore, we show that between-individual behavioural variation may play a role in forming landscapes of risk, and that population means may not be enough to identify these driving forces. The landscape of risk framework is most applied to interactions between predator and prey (although see [50,51]). However, competition is also a key ecological driving force. Extending the landscape of risk framework to non-predatory interspecific interactions provides opportunity to explore how behavioural responses to aggressive risk can cascade throughout communities.

Pairwise interactions represent a small part of a much larger complex network of interactions that shape one another [52], including competitive interactions and predation. For example, shared predators reduce competitive exclusion between prey species [52], and aggressive competitors can reduce clientele richness in cleaning interactions [53]. Although the impacts of competitive interactions on predation risk have been documented [47], there has been no attempt to model the spatial variation of predation and competition risk simultaneously. Incorporating multiple layers into landscapes of risk, representing various trophic levels, taxonomic groups and interspecific relationships will deepen our understanding of behavioural cascades through these complex interaction webs.

## Data Availability

All data and code are available in the electronic supplementary material. Raw video files and orthomosaics are available upon request. Supplementary material is available online [54].
